# Supplementary material for: Association between oral microbiome and seven types of cancers in East Asian population: a two-sample Mendelian randomization analysis
Source: Front Mol Biosci. 2023 Nov 21;10:1327893. doi: 10.3389/fmolb.2023.1327893 (PMC10702768; doi:10.3389/fmolb.2023.1327893)
Supplement: Supplementary file 1 [file DataSheet1.ZIP › Supplementary Materials/MR plots for tongue/tongue═╝/Colorectal cancer/pheno.2657_to_colorectal cancer_forest.pdf]

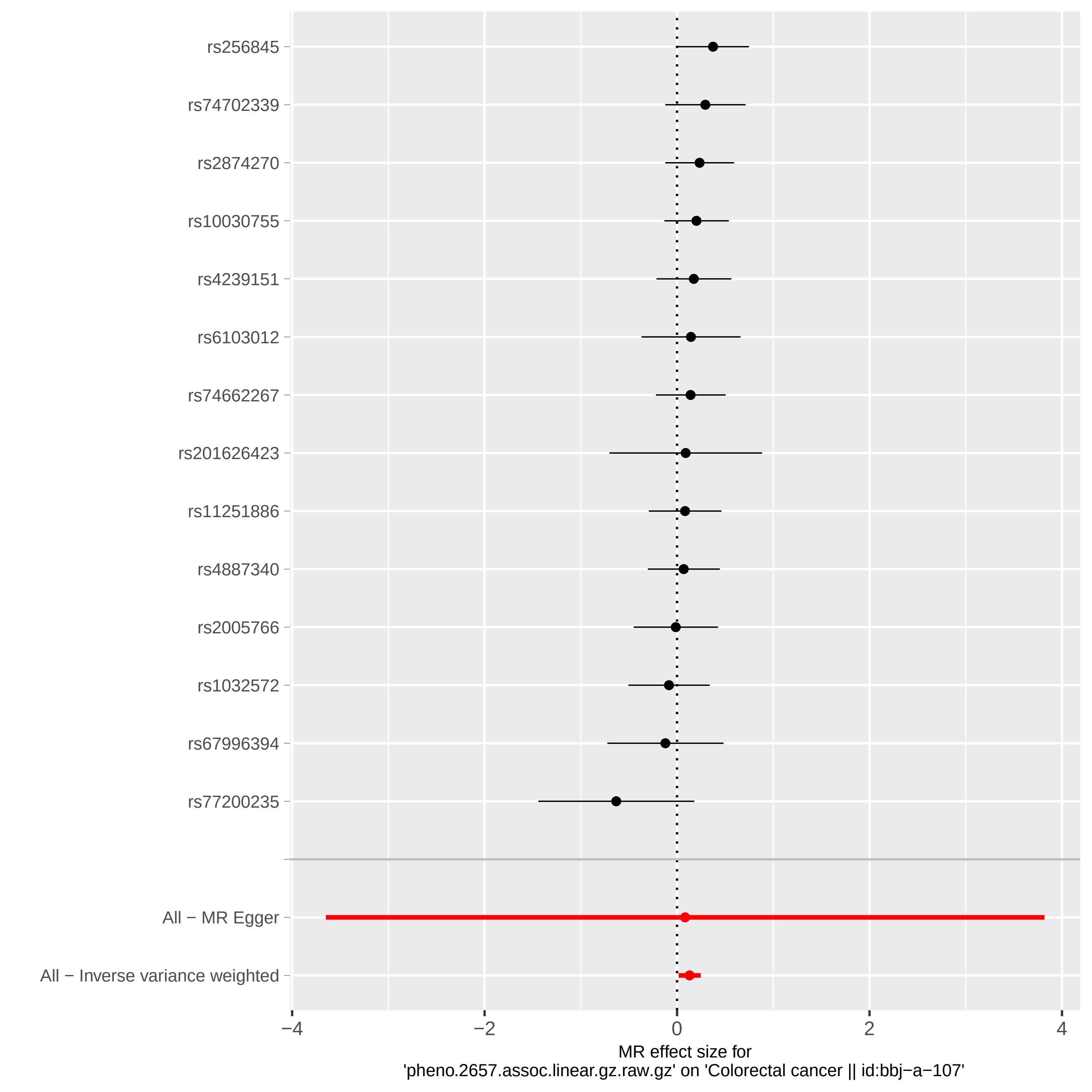

rs256845
rs74702339
rs2874270
rs10030755
rs4239151
rs6103012
rs74662267
rs201626423
rs11251886
rs4887340
rs2005766
rs1032572
rs67996394
rs77200235
All − MR Egger
All − Inverse variance weighted
−4
−2
0
2
4
MR effect size for
'pheno.2657.assoc.linear.gz.raw.gz' on 'Colorectal cancer || id:bbj−a−107'
